# Parent and Child Report of Pain and Fatigue in JIA: Does Disagreement between Parent and Child Predict Functional Outcomes?

**DOI:** 10.3390/children4020011

**Published:** 2017-01-30

**Authors:** Amy C. Gaultney, Maggie H. Bromberg, Mark Connelly, Tracy Spears, Laura E. Schanberg

**Affiliations:** 1 Duke Children’s Hospital and Health Center, 2301 Erwin Road, Durham, NC 27710, USA; 2 Seattle Children’s Research Institute, Center for Child Health, Behavior, and Development, M/S CW8-6, Seattle, WA 98145, USA; bromberg.maggie@gmail.com; 3 Children’s Mercy Hospital Kansas City, 2401 Gillham Road, Kansas City, MO 64108, USA; mconnelly1@cmh.edu; 4 Duke Clinical Research Institute, 2400 Pratt Street, Durham, NC 27705, USA; tracy.g.spears@duke.edu; 5 Duke Children’s Hospital and Health Center, 2301 Erwin Road, Durham, NC 27710, USA; laura.schanberg@dm.duke.edu

**Keywords:** pain, JIA, activity limitation

## Abstract

While previous research in juvenile idiopathic arthritis (JIA) has identified discrepancy between parent and child perception of disease-related symptoms such as pain, the significance and impact of this disagreement has not been characterized. We examined the extent to which parent-child discordance in JIA symptom ratings are associated with child functional outcomes. Linear regression and mixed effects models were used to test the effects of discrepancy in pain and fatigue ratings on functional outcomes in 65 dyads, consisting of youth with JIA and one parent. Results suggested that children reported increased activity limitations and negative mood when parent and child pain ratings were discrepant, with parent rated child pain much lower. Greater discrepancy in fatigue ratings was also associated with more negative mood, whereas children whose parent rated child fatigue as moderately lower than the child experienced decreased activity limitations relative to dyads who agreed closely on fatigue level. Implications of these results for the quality of life and treatment of children with JIA are discussed.

## 1. Introduction

Juvenile idiopathic arthritis (JIA) is a common chronic childhood illness often characterized by episodes of musculoskeletal pain and fatigue, which may contribute to problems with social and physical functioning [1,2]. Parents play an important role in the experience and treatment of pain and other symptoms in youth with chronic health issues [3]. Previous work has focused on the agreement between physicians and parents on the child’s global disease severity [4]; however, there has been limited research on whether discrepancies in ratings of pain and fatigue between parents and children with JIA influence social and physical activity participation and mood. In one study of children with JIA, Garcia-Munitis et al. demonstrated moderate to poor agreement on pain intensity ratings between parents and children, but did not identify predictors or outcomes of the reported discrepancy [5]. A longitudinal study by Palermo et al. examining parent-child discrepancy in pain reporting found that parents and children with JIA often disagreed on pain and functional disability ratings: the authors suggested a link between increased disagreement on pain ratings and increased child depression, but did not assess fatigue [6]. Their prior work is also limited in that it relied on one-time assessments or intermittent clinic based assessments, both subject to recall bias. Given the daily fluctuations in pain and fatigue typical of JIA, discrepancies in parent-child reports may be stable or dynamic over time. Intensive repeated measurement is ideal for assessing dynamic variables and characterizing reporting patterns [7]. This study employs an ecological data-gathering model to capture day-to-day variability in pain and fatigue reporting and interrogates whether the parent-child discrepancies themselves are associated with outcomes of interest.

The current study investigated discrepancies between parent and child reports of common JIA symptoms (pain and fatigue), expanding on the work of Garcia-Munitis et al. and Palermo et al. in describing discrepancies between parent report of child pain and fatigue and child self-reported fatigue in the setting of a daily electronic diary study. We also examined whether the direction of discrepancy affected the key outcomes of child negative mood and activity limitations. We expected to find that children whose parents over reported child pain and fatigue intensity would have poorer outcomes. This hypothesis is consistent with published literature suggesting that children whose parents use protective strategies, which might result in over reporting pain relative to the child, experience greater functional disability and negative mood [8,9,10].

## 2. Methods

The institutional review board at the study site approved study procedures (IRB Study ID: Pro00007325). Full study procedures were included in past publications using the larger dataset, which described child-reported daily symptom ratings and child sleep [11,12,13]. A pilot study performed with a smaller sample of patients examined parent responses to child pain [14]. The current study uniquely focused on the discordance between parent and child reports of pain and fatigue, not previously examined in this dataset. As part of the larger study, 74 dyads of children and their caregivers where initially recruited from the outpatient rheumatology clinic of an academic pediatric center. As the caregivers were predominantly biological mothers and fathers, with few other types of caregivers (stepparents, unspecified), we refer to the caregivers as “parents” for the remainder of the article. All children had a diagnosis of polyarticular JIA. Children were ineligible to participate if they had a current psychiatric diagnosis (specifically, mood disorders, fibromyalgia and pervasive developmental delay). The excluded disorders are known to affect pain and functioning and could confound experimental results. Children were also excluded if they were not attending school since school attendance was an outcome measure in the original study, physically incapable of completing the diary entries, non-English speaking, or if either they or the parent were illiterate. The sample analyzed for the current study includes 65 of the dyads; 9 dyads failed to complete the larger study or were missing data required for this analysis [15]. Of the included 65 dyads, 2537 fatigue reports (60.3% of all fatigue score reports) had both a parent and a child pain score, and 2411 pain reports had both a child and a parent pain score (57.3% of all pain score reports).

The dyads completed a battery of baseline questionnaires, including demographics reported by the parent. Each parent and child was provided with a T-Mobile Dash smartphone (T-Mobile, Bonn, Germany) and a study-specific instruction manual. Research staff trained each parent and child in use of the device. Each parent and child were instructed to complete thrice daily ratings of pain, fatigue, mood, and activity limitations at predetermined times selected by the family and programmed by the research staff. Diary data was collected for a total of 28 days.

### 2.1. Electronic Diary Variables

#### 2.1.1. Pain Intensity

**Figure 1:**
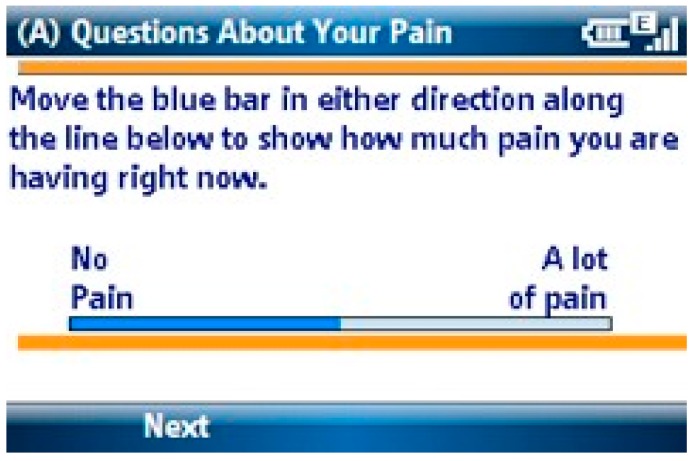
Pain Intensity Screen.

Children and parents rated pain intensity three times per day using a visual analogue scale (VAS) ranging from 0 to 100 mm based on a validated pain assessment for children [15]. Figure 1 shows the pain intensity VAS used.

#### 2.1.2. Fatigue Intensity

Children and parents were asked to rate the intensity of fatigue three times a day using a validated visual analogue scale (VAS) ranging from 0 to 100 mm [16].

#### 2.1.3. Activity Limitations

**Figure 2:**
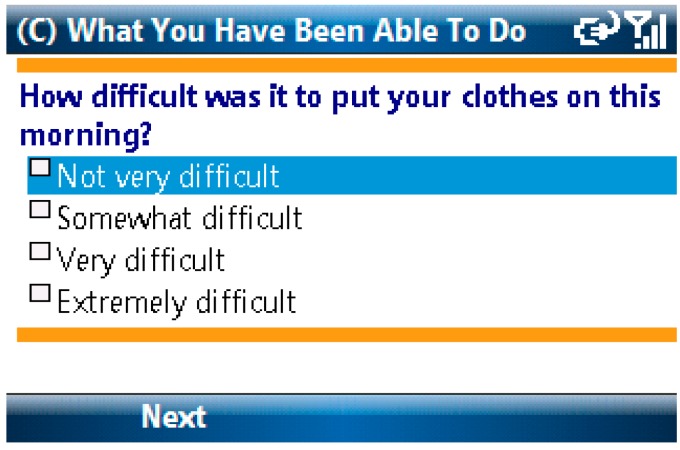
Activity Limitations Screen.

Items from the Activity Scale for Kids and the Child Activity Limitations Questionnaire were combined to assess physical, academic and social limitations in study participants. Children were asked to rate on a 4-point scale how difficult it was to complete each of eight different activities due to pain [17,18]. The list of activities differed depending on time of day. For example, the question “How difficult was it to put your clothes on this morning?” (See Figure 2) was asked only during the morning assessment. Other topics addressed in this scale included questions about difficulties with bathing, walking up stairs and staying seated in school. A total functional limitations score was calculated for each e-diary report by averaging the child’s responses.

#### 2.1.4. Negative Mood

Child self-reported negative mood was measured by 5 items taken from the Positive and Negative Affect Scale for children (PANAS-C), which was initially validated in a general population of school-aged children [19]. Responses to the negative affect items were averaged to provide a mean negative mood score on each e-diary report. Research in adults and the school-aged population has demonstrated that negative affect scores are significantly correlated with symptoms of both anxiety and depression [19,20]. The initial scale validators argue that the negative mood score is best seen as a measure of psychological distress [20].

### 2.2. Data Analysis Plan

**Figure 3:**
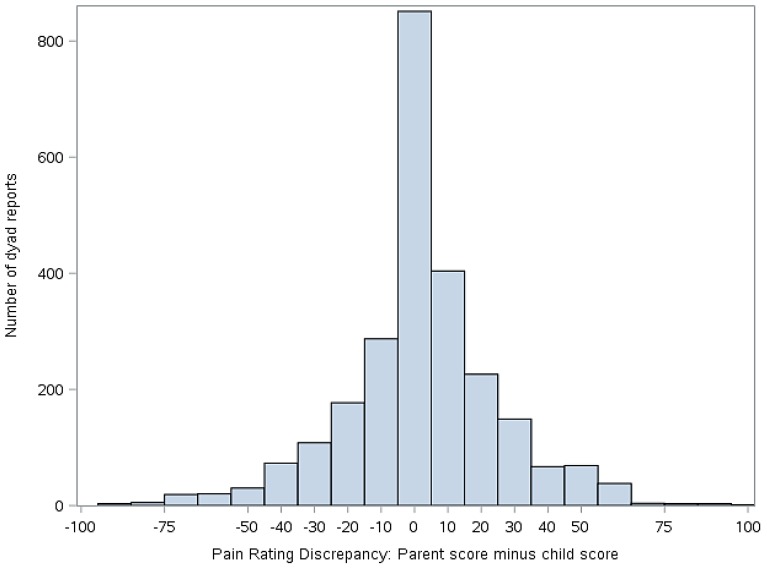
Discrepancy between child and parent ratings of child pain (parent score minus child score). Positive values indicate that the parent scored pain higher than the child. Negative numbers indicate that the child scored pain higher than the parent.

**Figure 4:**
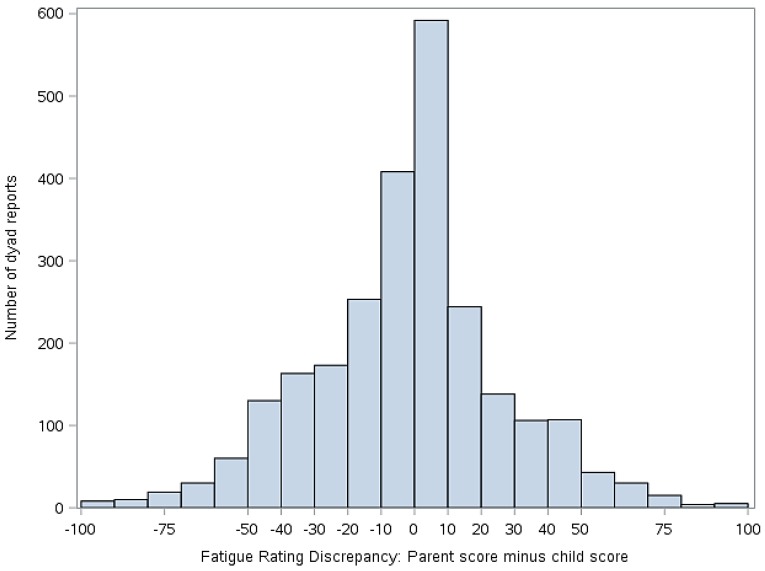
Discrepancy between child and parent rating of child fatigue (parent score minus child score). Positive values indicate that the parent scored the fatigue higher than the child. Negative numbers indicate that the child scored the fatigue higher than the parent.

Statistical analyses were performed using version 9.4 of Statistical Analysis Software (SAS) (Cary, NC, USA). Descriptive statistics (means, standard deviations, frequencies) were used to summarize demographics and primary study variables, as applicable. To address our primary aim, mixed effect models were constructed to evaluate the extent to which parent-child discordance in symptom ratings (pain and fatigue) were associated with the two outcomes of interest (activity limitations and mean negative mood score). To derive the predictor variable for these analyses, we first calculated simple difference scores (parent minus child report) on ratings of pain and fatigue. The resulting discrepancy scores then were classified into five groups for analyses, as follows: −100 to −51 mm (high discrepancy, parent < child), −51 to −11 mm (moderate discrepancy, parent < child), −10 to 9 mm (low discrepancy), 10–49 mm (moderate discrepancy, parent > child), and 50–100 mm (high discrepancy, parent > child). The decision to group the scores was made to facilitate data interpretation and presentation as well as to clearly delineate whether the extent and direction of disagreement were correlated with functional outcomes. The cut points for the groups were selected on the basis of the data distribution shown in Figure 3 and Figure 4. The parent-child discrepancy groups were treated as fixed effects, and the parent-child dyad was specified as a random effect to allow clustering of repeated measures by dyad. The low discrepancy group (score discrepancies of −10 to 9 mm) was used as the reference group in all analyses. Reference cell coding was used for the discrepancy groups. Analyses were adjusted for days since study onset (fixed effect), since it was associated with our outcomes of interest (activity limitations and mean negative mood score).

## 3. Results

### 3.1. Descriptive Statistics

#### 3.1.1. Demographics

Our sample was predominantly female (72% female) and Caucasian (75% Caucasian, 19% African American, 5% unanswered and 1.5% Pacific Islander). The mean age of children in our sample was 12.7 years, with a standard deviation of 2.8 years.

#### 3.1.2. Discrepancies between Child and Parent Report of Pain and Fatigue

Figure 3 illustrates the distribution of the simple difference between parent and child pain scores, while Figure 4 illustrates the distribution of simple difference between parent and child fatigue score at all study time points. “Simple difference” refers to parent score minus child score. For example, if a parent rates the child’s pain at 50 mm and the child rates their pain at 75 mm, the simple difference is −25 mm. For Figure 3 and Figure 4, when *x* = 0, there is no difference between parent and child score. The *y* axis represents the number of dyad reports in each group over the course of the study.

Table 1 and Table 2 provide the distribution of simple difference between parent and child pain and fatigue scores, respectively, for all time points in the study. The percent recorded in the second column represents the percentage of all observations in the study that fall into each discrepancy group, where *N* = total study observations.

### 3.2. Hypothesis Testing

#### 3.2.1. Parent-Child Discrepancies in Pain Intensity and Fatigue Reports as a Predictor of Mood

Compared to the low discrepancy reference group, children whose parents over reported their child’s pain by 50–100 mms had a higher mean negative mood score (*p* value = 0.0136), as shown in Table 3. Children whose parents either over reported the child’s fatigue by 50–100 mms or under reported their child’s pain by 11–50 mms also had higher negative mean scores (*p* value = 0.003 and 0.002, respectively), as shown in Table 4.

#### 3.2.3. Parent-Child Discrepancy in Pain Intensity and Fatigue Reports as a Predictor of Activity Limitation

Children whose parents under reported their pain by 49 to 100 mms had a higher activity limitation score (*p* = 0.043) compared to the low discrepancy group, as shown in Table 5. Conversely, children whose parents under reported their fatigue by 11 to 50 mms had fewer activity limitations (*p* = 0.016), as shown in Table 6.

## 4. Discussion

This study examined associations between degree of discordance in parent and child ratings of child symptoms, functioning, and mood. Our data suggest that parent reports of child pain and fatigue often closely match those of the child, as evidenced by the center clustering in Figure 3 and Figure 4. However, we also found that parents and children at times disagreed markedly on these measures and that discrepancies in reports, when present, were correlated with functional outcomes.

In contrast with what was expected, we found that children whose parents highly underreported their pain experienced higher rates of negative mood and greater activity limitation. We hypothesized that children whose parents overreported their pain would have greater activity limitations and negative mood based on previous research regarding parental overprotectiveness and its impact on activity and mood. However, we did not find a relationship between parental over reporting of child pain and increased negative mood or activity limitations.

It is possible that parents under report their child’s pain as a minimizing response. Children may then respond by experiencing and recording an exaggerated pain level in an attempt to legitimize their experience, which would further exacerbate parent-child discordance. Previous work in children with chronic pain has demonstrated that minimizing parental responses can contribute to increased somatic symptoms, particularly in anxious children [21]. Additionally, previous work has demonstrated that depression in JIA patients is associated with higher activity limitation and higher disease severity [22]. Although negative mood is not synonymous with depression, as a marker of general psychological distress, it may be that negative mood plays a synergistic role with parental discordance in driving activity limitations [19].

We also found that children whose parents moderately overreported or highly underreported their fatigue had increased negative mood. Children whose parents underreported their fatigue had lower levels of activity limitation, but we again did not find that parental overreporting of fatigue correlated with greater activity limitation in the child. Our data regarding fatigue and negative mood suggest that child-parental discordance itself regarding perception of fatigue is correlated with greater negative mood in the child. It is unclear from our study why discrepancy in fatigue reporting generally, rather than discrepancy in a particular direction (e.g., parental over- or under reporting), was correlated with increased activity limitation. It may be that our measurement of discrepancies in parent and child fatigue report is confounded by a lack of family cohesiveness or poor communication, which drives both bidirectional disagreement on fatigue level and increased negative mood in the child. Previous research has demonstrated an association between a negative family environment and depressive symptoms in healthy children [23]. Our findings regarding parent-child discrepancy in fatigue report contributes to a gap in knowledge regarding fatigue in JIA. Although we know that fatigue is common in JIA, there is significantly less published work on fatigue in this disease relative to pain, and the existing evidence is somewhat conflicting [14,24,25].

Future work should explore whether particular family behavioral patterns are associated with increased disagreement between parent and child reports of symptoms and functional outcomes, data which was not available to us in this cohort. The current findings could be further characterized by future research exploring whether different caregiver characteristics predict discordance with child symptom report and whether different caregivers of the same child show agreement on child pain and fatigue ratings. Caregivers participating in this study were not diverse enough to explore inter-rater agreement between children and different types of caregivers, such as grandparents, mothers and fathers. Garcia-Munitis et al. demonstrated that certain caregivers, namely mothers, are more likely than others to agree closely with the child [5]. Additional family factors such as communication quality and style and level of parental monitoring may also be related to discordant reporting; future work should evaluate the role of these factors. It would also be worthwhile to explore whether informing the parent and child of the difference in their perception of the child’s pain and fatigue would improve agreement between their ratings, and ultimately, the child's pain, mood, and ability to participate in social and physical activities.

Our results should be interpreted in light of several limitations. While the results discussed were statistically significant, we cannot infer causation. We view this work as preliminary and as a starting point for further research. A type one error is always a possibility given that we ran several statistical analyses. The sample was obtained from a single center and was a sample of convenience, limiting generalizability. All measures of pain and fatigue were self-reported, and some children may be poor or inaccurate reporters. In addition, unaccounted for confounders may have influenced the results.

In spite of these limitations, these results suggest that discrepancy often exists between parent and child ratings of pain and fatigue, raising questions about how clinicians respond to parent and child report of symptoms when there is considerable discrepancy, especially considering treatment decisions. There are objective measures of disease activity in JIA, such as inflammatory markers, joint exam, X-rays, but subjective patient and parent reported symptoms also guide therapy. This study reaffirms the need for clinicians to assess perception of disease severity from the perspective of both parent and child when making treatment decisions. Additionally, because the results suggest that the discrepancies are associated with functional outcomes, healthcare providers may preemptively wish to refer parents and children who demonstrate disagreement on levels of pain and fatigue to mental health professionals such as social workers and psychologists, as these children may be at risk for suboptimal functional and emotional outcomes.

## Figures and Tables

**Table 1:** Groups of Pain Score Discrepancy.

Discrepancy Group (Parent Score-Child Score)	Dyad Reports *n* (%) (*N* ^1^ = 2537)
High Discrepancy (−100 to −51, child rated higher)	177 (7.0%)
Moderate Discrepancy (−50 to −11, child rated higher)	467 (18.4%)
Low Discrepancy (−10 to 9, parent and child rated similarly) ^2^	1172 (46.2%)
Moderate Discrepancy (10 to 49, parent rated higher)	640 (25.2%)
High Discrepancy (50 to 100, parent rated higher)	81 (3.2%)

*^1^ N* represents the total number of fatigue reports for the entire study. Percentages represent the number of individual reports in each group; ^2^ Reference group in mixed model analysis.

**Table 2:** Groups of Fatigue Score Discrepancy.

Discrepancy Group (Parent Score-Child Score)	Dyad Reports *n* (%) (*N* ^1^ = 2411)
High Discrepancy (−100 to −51, child rated higher) ^2^	0
Moderate Discrepancy (−50 to −11, child rated higher)	719 (29.8%)
Low Discrepancy (−10 to 9, parent and child rated similarly) ^3^	1000 (41.5%)
Moderate Discrepancy (10 to 49, parent rated higher)	595 (24.7%)
High Discrepancy (50 to 100, parent rated higher)	97 (4.0%)

^1^ There were no dyads reports in the −100 to −49 range (parent fatigue score was much lower than child score); ^2^ *N* represents the total number of fatigue reports for the entire study. Percentages represent the number of individual reports in each group; ^3^ Reference group in mixed model analysis.

**Table 3:** Relationship between simple difference of pain score and mean negative mood score.

Predictor variable: Discrepancy Group ^1^ (Parent Score-Child Score, Pain)	Estimate	Standard Error	*p*-Value
High Discrepancy (−100 to −51, child rated higher)	0.074	0.030	0.014
Moderate Discrepancy (−50 to −11, child rated higher)	0.001	0.020	0.964
Moderate Discrepancy (10 to 49, parent rated higher)	0.017	0.019	0.373
High Discrepancy (50 to 100, parent rated higher)	0.011	0.045	0.813

^1^ The reference group in mixed model analyses (not shown) is the low discrepancy group.

**Table 4:** Relationship between simple difference of fatigue scores and mean negative mood scores.

Predictor Variable: Discrepancy Group ^1^ (Parent Score-Child Score, Fatigue)	Estimate	Standard Error	*p*-Value
High Discrepancy (−100 to −51, child rated higher) ^2^	-	-	-
Moderate Discrepancy (−50 to −11, child rated higher)	0.056	0.017	0.002
Moderate Discrepancy (10 to 49, parent rated higher)	0.016	0.019	0.397
High Discrepancy (50 to 100, parent rated higher)	0.113	0.038	0.003

^1^ The reference group in mixed model analyses (not shown) is the low discrepancy group; ^2^ There were no dyads in the −100 to −49 range (parent fatigue score was much lower than child score).

**Table 5:** Relationship between simple difference of pain scores and activity limitation.

Predictor Variable: Discrepancy Group ^1^ (Parent Score-Child Score, Pain)	Estimate	Standard Error	*p*-Value
High Discrepancy (−100 to −51, child rated higher)	1.097	0.538	0.043
Moderate Discrepancy (−50 to −11, child rated higher)	0.277	0.368	0.452
Moderate Discrepancy (10 to 49, parent rated higher)	−0.037	0.354	0.916
High Discrepancy (50 to 100, parent rated higher)	−1.471	0.820	0.075

^1^ The reference group in mixed model analyses (not shown) is the low discrepancy group.

**Table 6:** Relationship between simple difference of fatigue scores activity limitation.

Predictor Variable: Discrepancy Group ^1^ (Parent Score-Child Score, Fatigue)	Estimate	Standard Error	*p*-Value
High Discrepancy (−100 to −51, child rated higher) ^2^	-	-	-
Moderate Discrepancy (−50 to −11, child rated higher)	−0.796	0.328	0.016
Moderate Discrepancy (10 to 49, parent rated higher)	−0.230	0.350	0.512
High Discrepancy (50 to 100, parent rated higher)	−1.179	0.704	0.096

^1^ The reference group in mixed model analyses (not shown) is the low discrepancy group; ^2^ There were no dyads in the −100 to −49 range (parent fatigue score was much lower than child score).
